# Iterative Learning Control for a Soft Exoskeleton with Hip and Knee Joint Assistance

**DOI:** 10.3390/s20154333

**Published:** 2020-08-04

**Authors:** Chunjie Chen, Yu Zhang, Yanjie Li, Zhuo Wang, Yida Liu, Wujing Cao, Xinyu Wu

**Affiliations:** 1 CAS Key Laboratory of Human-Machine-Intelligence Synergic Systems, Shenzhen Institutes of Advanced Technology, Shenzhen 518055, China; cj.chen@siat.ac.cn (C.C.); 18s053220@stu.hit.edu.cn (Y.Z.); zhuo.wang@siat.ac.cn (Z.W.); yd.liu1@siat.ac.cn (Y.L.); wj.cao@siat.ac.cn (W.C.); 2 Guangdong Provincial Key Lab of Robotics and Intelligent System, Shenzhen Institutes of Advanced Technology, Chinese Academy of Sciences, Shenzhen 518055, China; 3 ShenZhen College of Advanced Technology, University of Chinese Academy of Sciences, Shenzhen 518055, China; 4 Harbin Institute of Technology, School of Mechanical Engineering and Automation, Shenzhen 518055, China; autolyj@hit.edu.cn

**Keywords:** lower limb assistance, soft exoskeleton, iterative learning control, force tracking, metabolic cost

## Abstract

Walking on different terrains leads to different biomechanics, which motivates the development of exoskeletons for assisting on walking according to the type of a terrain. The design of a lightweight soft exoskeleton that simultaneously assists multiple joints in the lower limb is presented in this paper. It is used to assist both hip and knee joints in a single system, the assistance force is directly applied to the hip joint flexion and the knee joint extension, while indirectly to the hip extension also. Based on the biological torque of human walking at three different slopes, a novel strategy is developed to improve the performance of assistance. A parameter optimal iterative learning control (POILC) method is introduced to reduce the error generated due to the difference between the wearing position and the biological features of the different wearers. In order to obtain the metabolic rate, three subjects walked on a treadmill, for 10 min on each terrain, at a speed of 4 km/h under both conditions of wearing and not wearing the soft exoskeleton. Results showed that the metabolic rate was decreased with the increasing slope of the terrain. The reductions in the net metabolic rate in the experiments on the downhill, flat ground, and uphill were, respectively, 9.86%, 12.48%, and 22.08% compared to the condition of not wearing the soft exoskeleton, where their corresponding absolute values were 0.28 W/kg, 0.72 W/kg, and 1.60 W/kg.

## 1. Introduction

As people age, walking efficiency declines because step width increases and stride length decreases [1]. In order to provide assistance for the older adults, the lower extremity exoskeleton robot, a wearable device for assisting humans in walking and running, has been developed widely in the past few decades. The majority of lower limb exoskeletons have a rigid structure for ensuring high assistive force to the wearer at the time of carrying a load, it is successfully applied to the rehabilitation of patients without mobility [2]. However, a rigid structure restricts freedom and affects the comfort of wearers [3]. Because of such drawbacks with rigid structures, many researchers have focused their attentions on soft exoskeletons and designed many soft wearable devices. A soft exoskeleton assisting both upper and lower body is designed in [4], and the Myosuit is developed to reduce the hip extensor activity in sitting transfers [5]. A research group from Harvard University have designed a series of exosuits, where a textile is used to exert an assisting force parallel to the hip muscles or ankle joint, or parallel to both of them [6,7,8]. The Xosoft EU project designed a series of soft exoskeleton through advanced textiles and smart materials, which can provide assistance according to user’s motion and intention [9,10]. Other researchers apply Pneumatic Muscle to exert assistive forces to different joints [11,12,13,14]. All such designs aim to augment the musculature by providing assistive force parallel to muscles, which are mainly used to assist the elderly in their daily life [15,16]. In comparison to a rigid structure, a soft exoskeleton has other advantages also, such as lightweight, high flexibility, better comfort, and so on.

Although a soft material can improve comfort and flexibility for a wearer, it also increases the complexity of the design of the mechanism and controller used in the exoskeleton. Because of the ductile structure, a soft exoskeleton system has numerous uncontrollable degrees of freedom and a more complex model to fabricate [17], which is a big challenge to control a soft exoskeleton. The reference profile approach is often used to generate asssitive force by controlling the motor position [7,18,19,20]. However, this method lacks robustness and fails to track the reference force profile accurately. The adding admittance control is used in the controller approach for reducing the error in tracking the desire force profile [21]. However, it is still difficult to develop a precise model of the motor required in this approach. As human walking is a repetitive process, a soft exoskeleton needs to control it accordingly. However, the above methods do not take into account the repetitive disturbance in the gait cycle. In order to improve the tracking performance, the iterative learning control (ILC) utilizes error history from the previous control cycle [22]. This strategy has been successfully used in some soft robotics. The combination of ILC and low-gain feedback control approach is used in [23] to reduce the tracking error. An ILC-based method is reported in [24] to learn the grabbing tasks in a soft fluidic elastomer manipulator. In [25], a norm-optimal iterative learning control scheme, based on a liner model, is used to improve the position tracking performance of a soft robotic arm. However, such applications of ILC did not consider the problem of control cycle and control target changing, which are inevitable in any soft lower limb exoskeleton. In [26], the tracking error was reduced by altering the fixed position based on previous error. Although this method provided decent tracking of the desired profile, its performance suffered from a slow rate of convergence once tuning the desired profile. The reinforcement learning methods have been used in robots [27,28]; however, these methods are not applicable to soft exoskeleton systems because of the complex calculation. In order to improve the performance of soft exoskeleton in terrain with different slopes, a novel parameter optimal iterative learning control (POILC)-based controller is designed in this paper. The controller can determine the terrain according to the angles of lower limb motion, and then choose different assistance strategies according to the terrain. Meanwhile, the POILC method is used to decrease the tracking error.

Another challenge in a soft lower limb exoskeleton is to determine suitable assistive forces at different times in the gait cycle. The changes in torques in hip, knee, and ankle joints at different time in the gait cycle during walking on the ground were studied in [29,30]. Based on those results, the assistive force profile was generated by combing two independent parameterized sinusoidal curves [21,31]. The strategy developed from flat ground to control the exoskeleton fails to work properly on uneven terrain. As an enhanced exoskeleton can often operate on different terrains, it is necessary to study the changes in the torque on the lower limb joint during walking on uneven ground. The changes in the mechanism of the lower limb joint during walking on uneven terrain was studied in [32], where it was found that the torque in the knee joint increased significantly on sloped terrain. However, very few researchers have so far referred this result in the design of exoskeleton assistance strategies on uneven terrain. In order to frame a proper assistance strategy, human biological information is considered to verify the parameters of the control strategy through the Covariance Matrix Adaptation Evolution Strategy (CMA-ES) or the Bayesian optimization [31,33,34]. These methods are not suitable to design an exoskeleton controller that will need to get adapted to different terrains at outdoor environments. This is because the slow convergence rate and the wearing of a breathing mask restrict the application of an exoskeleton. In this paper, three assistance strategies for three slopes are designed according to the biological moment of human walking on corresponding terrains.

Many researchers studied multi-joint assistance for the soft lower limb [16] and designed strategies to assist both the hip and knee extension simultaneously [35]. In this paper, the assistances of hip and knee joints are combined to provide assistance to the hip joint flexion and extension, as well as to the knee joint extension. The hip and knee joint extension assistance in different terrains with the same assistance strategy is realized in our previous work [36]; in this paper, we propose a novel assistance scheme of soft exoskeleton to improve the performance of assistance on uneven terrain. The advantage of this novel scheme is that a single actuator can be used to provide assistance force to both hip and knee joints, which reduces the system weight and makes the system more convenient to wear. In order to provide the proper assistance torque in our system, we build a novel assistance strategy based on the moments of the hip and knee joints on the gait cycle on different terrains. This paper also presents a robust control strategy with application for parameter optimization in the iterative learning control, which improves the control performance. The major contributions of this paper are as follows.

1. A novel assistance scheme is proposed for assisting one-leg hip joint flexion and extension as well as knee joint extension through a single actuator, which is obtained by actuating the Knee Board and the wraps of the front side of the hip with one motor. It is beneficial to decrease the system’s weight and increase the convenience of wearing. The muscle fatigue of wearers can be reduced significantly through this scheme on different terrains.
2. We employ an iterative learning control approach, based on parameter optimization, to improve the performance of assistance by compensating the error generated from the wearing position and terrain change. The approach makes the exoskeleton suitable for different pilots.
3. The assistance strategy is built based on the biological moment of human walking on different slopes, and the resulting effectiveness is verified through experiment.

The rest of the paper is organized as follows. The detailed design of the soft exoskeleton is explained in Section 2 along with its movement characteristics and a simple model. The assistance strategy and the algorithm of control are discussed in Section 3. The experimental results and analysis are presented in Section 4. Our soft exoskeleton is discussed in Section 5. Finally, a conclusion is drawn in Section 6.

## 2. System Overview

Our designed soft exoskeleton can provide assistance force to hip joint extension and flexion and also to knee joint extension. It is used mainly to augment the musculature of healthy adults. It aims to increase human endurance during walking or running. In order to minimize the weight of the system and to increase the convenience of wearing, we intended to design our exoskeleton in a highly integrated manner.

### 2.1. Design of The Proposed Soft Lower Limb Exoskeleton

To understand the characteristics of the joint torque in the lower limb when walking on different terrain, McIntosh et al. [30] studied the changes in the moment of the lower limb joint at different terrain inclination angles. The results show that the torques in the hip and knee joints have a big difference at different terrain inclinations, which implies that providing assistance to the knee joint is beneficial to reduce the fatigue of the muscle. Hip flexion and hip extension are two separate phases at different times in the same gait cycle [29], which means that we can provide assistance to hip extension and hip flexion through a single actuator motor. Asbeck et al. [19] also found that this method is beneficial to reduce the muscle fatigue of wearers. Therefore, we designed a novel soft exoskeleton to provide assistance to hip joint extension and flexion and knee joint extension through a single actuator motor, so as to improve the performance of the soft exoskeleton.

**Figure 1:**
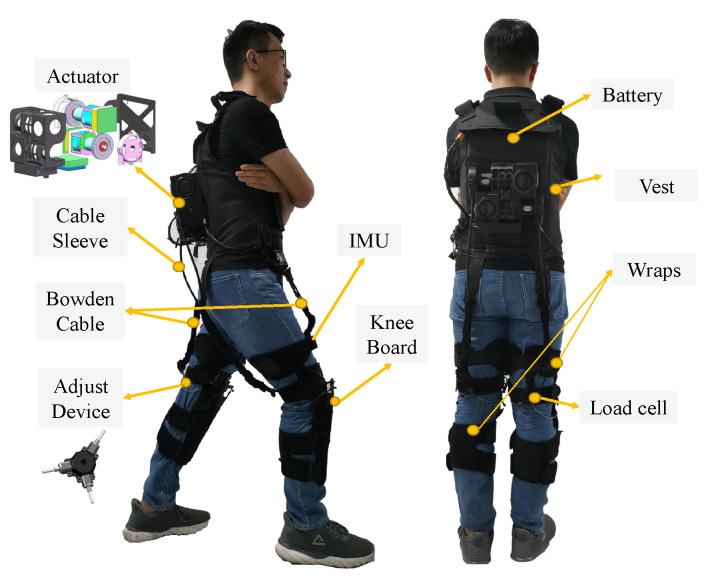
The system overview showing the structure of the soft exoskeleton and the position of each of its parts.

Our soft exoskeleton system comprises six parts on each leg: actuation module, Bowden cable, a Bowden cable adjusting unit, load cells, Inertial Measurement Unit (IMU) sensor, and soft wraps as shown in Figure 1. There are four fixing points on the vest and three on the leg wrap of each leg connected through the Bowden cable as shown in Figure 1. One end of the Bowden Cable is connected to the motor through Cable sleeve, and the other end is connected to Adjust Device. The part of the Bowden Cable exposed in the middle is wrapped by soft textiles to separate the signal cable and Bowden Cable. Upon actuating the motor, a tensile force is generated in the Bowden cable, which produces a torque at the fixing point. The initial length of the Bowden cable can be quickly adjusted according to the height of the wearer through its adjusting unit. The IMU sensor (WT901C485, Shenzhen wit-motion Technology, China), installed on the wraps of the hip, is used to collect data related to the angle, velocity, and acceleration of the thigh movement. The load cell (GJBLS-WS, Bengbu Zhongcheng Sensor, China), whose one end is connected to the Bowden cable and the other end to the fixing point, is used to monitor the change in the force in real-time and then to return the force data to the micro-controller. Other components of our soft exoskeleton include a nylon vest used to fix the actuation system, two binds to wrap the subject’s hip, and two Knee Boards (carbon fiber boards) to transmit the extension torque to the knee and hip joints.

**Figure 2:**
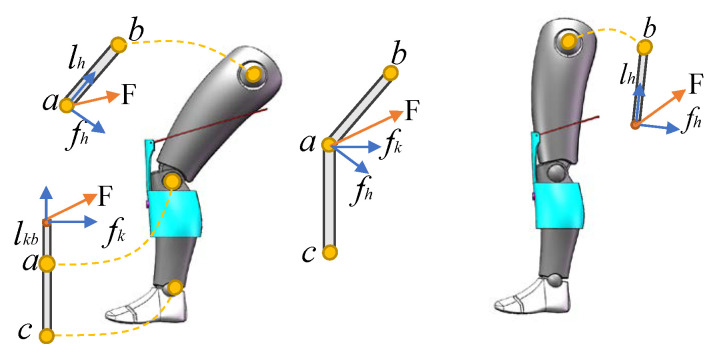
The two states of the Knee Board. Where *a* and *b* represent the knee and hip joint center respectively; $l_{h}$ and $l_{kb}$ represent the length of thigh and Knee Board, respectively; *F* is the tension in Bowden cable; and $f_{h}$ and $f_{k}$ represent the component forces in the hip and knee joints, respectively, which can produce the joint moment.

Co-assistance for the hip and knee joints is achieved through the Knee Board, which is an innovative design for the soft exoskeleton system, as shown in Figure 2. The forces transmitted to the Knee Board through the Bowden cable can be decomposed into a component in the vertical direction and another component in the parallel direction of the Knee Board, and then the vertical force component will produce the torque in the knee joint. Considering the Knee Board and the shank as a whole, we can decompose the tension in the Bowden cable into a component in the vertical direction and another component in the parallel direction of the thigh. Then, the force component vertical to the thigh will produce the torque in the hip joint. These are shown in the left part of Figure 2, panels (a) and (b) represent the knee and hip joint center, respectively. *F* is the tension in Bowden Cable; $f_{h}$ and $f_{k}$ are the force components in the hip and knee joint, respectively, which produce assistance moment; and $l_{h}$ and $l_{k}$ are the length of the thigh and Knee Board, respectively. The relationship between the force of the Bowden Cable and the assistance force of hip and knee joint is shown in the middle of Figure 2. When the legs are straight, as shown in the right part of Figure 2, the force applied to the Knee Board generates only moments in the hip joint.

The soft exoskeleton we designed is a system with two degrees of freedom and it is capable of assisting the flexion and extension of the hip joint and the extension of the knee joint during walking or running on both flat ground and slope. Each actuation module consists of a Brushless Motor (MG-1/S 6010, DJI, Shenzhen, China) connected directly to a planetary gearbox of ratio of 16:1 to drive an aluminum alloy pulley having diameter of 73 mm. The model of our adopted motor is ADM-15D80-CALT (ADM-15D80-CALT, Techservo, Shenzhen, China) and the maximum and continuous torque are 3.2 N·m and 1.5 N·m, respectively, which communicates with a microprocessor through the CAN-Open communication protocol. The microprocessor (STM32F407, STMicroelectronics, Milano, Italy) is used to process the data collected by the IMU and the load cells, and then to send commands for the required position to the motor actuator. To improve the efficiency of the control system, we run the control algorithm in the Raspberry Pi 4B (Raspberry Pi 4, Sony, Pencoed, Wales) and communicate the results with the microprocessor through a serial port. The system is powered by a Li-ion battery with a voltage of 48 V and a capacity of 3 Ah.

Knapik et al. [37] demonstrated that the energy cost can be minimized by displacing the center of mass (COM) of the load as close as possible to the COM of the body. Therefore, 69% weight of our soft exoskeleton is put on the back, at a position close to the center of mass of the body, so as to minimize the consumption of additional oxygen due to the weight of the exoskeleton. The total weight of our exoskeleton is 4859g. The weight of each part are given in Table 1.

### 2.2. Movement Character

**Figure 3:**
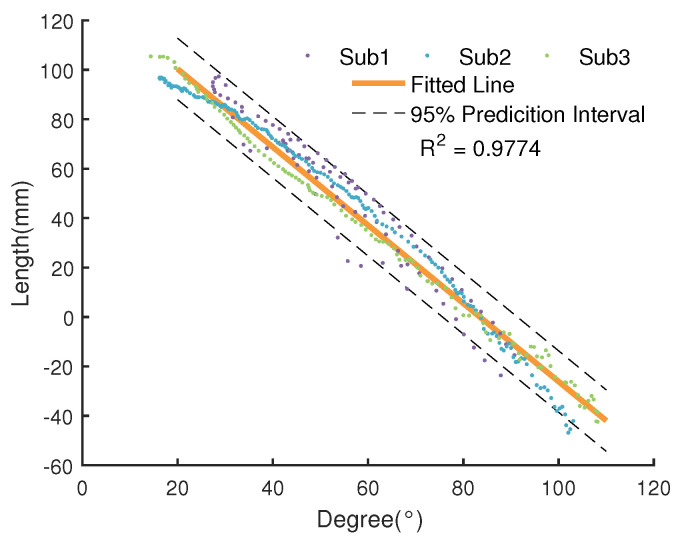
Relationship between the angle of hip joint and the length of Bowden cable. Where the angle of 0 represents the thigh is perpendicular to upper body, the position of 0 represent the position of Bowden Cable when the lower limb remains upright, the $R^{2}$ is the coefficient of determination.

The length of the Bowden cable gets changed with the motion of the thigh, which disturbs the control of the exoskeleton system. We were able to reduce this effect by adding a feedforward unit, which was built based on the relationship between the length of the Bowden cable and the hip angles. The movement of lower limbs when human walks can be approximated as rotation in the sagittal plane. Meanwhile, as the upper body has been kept vertical during walking, we can obtain the hip angle with respect to the vertical plane from a single IMU attached on the thigh. In order to derive this relationship, we conducted experiments with three healthy male subjects (24±1 years old; 66±3 kg weight; 176±5 cm height). The hip angles and the length of the Bowden cable were measured through the IMU and the motor encoder, respectively. We recorded the hip angles from the IMU and the length of the Bowden cable when the thigh was swinging slowly. A tension of 5 N was applied to keep the Bowden cable tight in order to acquire the length of the Bowden cable through the encoder. The obtained results are shown in Figure 3.

The splattering in the picture are the raw data of the subjects, and the blue line is the model of the thigh motion as expressed by Equation (1).

(1) $$\begin{array}{c} l=a\theta +b \end{array}$$

where θ is the hip angle; *l* is the length of Bowden cable; *a* and *b* are two coefficients of this model, whose values are obtained through curve fitting as −1.5808 and 131.9565, respectively. It is noteworthy that the relationship between the length of the Bowden cable and the hip angle is similar for both front and back of the hip. Therefore, for the sake of simplicity, the data in this section are taken from the back side of the hip joint.

### 2.3. Modeling

**Figure 4:**
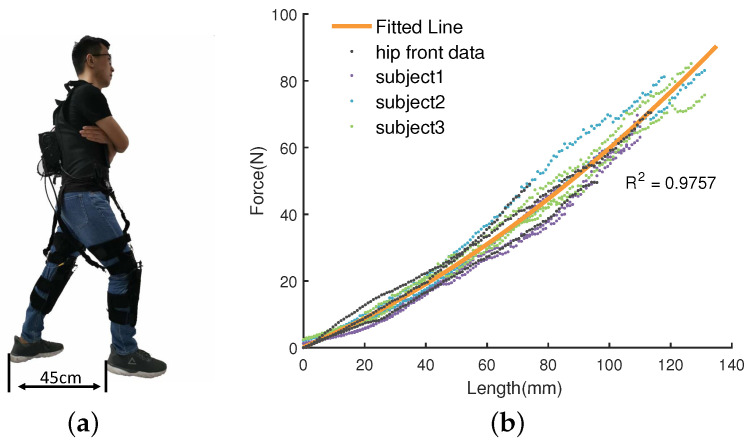
The stiffness model of the soft exoskeleton. Where (**a**) is the standing posture for experimenter, and the (**b**) is the result. The experiment is conducted by retracting the Bowden cable while holding the initial standing posture. The splatting is the raw data from experiment and the black dash line denotes the data that were implemented in the front of hip joint. The blue line is a fitted line with a coefficient of determination of 0.9757.

In order to understand the dynamics of the soft exoskeleton in our system, we develop a relationship between the tension and the length of the Bowden cable based on a series of experiments conducted on the same subjects. The experimenters wear an exoskeleton and stand on the flat ground while keeping a 45 cm distance between their feet. The experiment subjects wear an exoskeleton and stand on the flat ground while keeping a 45 cm distance between feet, as shown in Figure 4a. The actuator retracts the Bowden cable while holding the initial standing posture when actuator retracts Bowden cable to analyze the relationship between the change in the length and the tension of the Bowden cable. The sampling time was set as 0.01 s. The initial tension in the Bowden cable was made 2 N and its initial length was recorded as 0 mm. In order to ensure the safety of the subjects, we restricted the maximum tension and the maximum length to within 150 N and 150 mm, respectively. We performed the experiments in both front and back sides of the hip joint. The experimental conditions were kept the same in both front and back sides, except that the posture of the legs was reversed.

Figure 4 shows the relationship between the length of the Bowden cable and the tension in it. The black colored dash line shows the data of the front side of the hip, whose distribution remains same in its back side. Therefore, it is reasonable that we build the relationship between the length and the tension of the Bowden cable based on the experiment conducted in the backside of the hip. The yellow line is the stiffness model which can be expressed mathematically as given by Equation (2).

(2) $$f=a_{1}l^{2}+a_{2}l+a_{3}$$

where *l* and *f* represent, respectively, the length and the tension of the Bowden cable, and $a_{1}$, $a_{2}$, and $a_{3}$ are the coefficients of this mode, whose values are 0.0021, 0.3908, and 0.1883, respectively.

The dynamics of the soft exoskeleton are assumed to be driven by the length of the Bowden cable which are changed through the actuation of the motor, enabling us to model the dynamic system based on the relationship between the position of the motor and the tension in the Bowden cable. Due to the flexibility of the material and other uncertain parameters, it is a big challenge to model the dynamic system directly. To overcome this problem, we used the system identification method to build the dynamic model of the soft exoskeleton. We used the length of the Bowden cable as the input (Δl), and obtained the tension in the Bowden cable (*f*) as the output. Other input data were obtained from the experiment. We used the toolbox of system identification in MATLAB to build the dynamic model. The identification result is that the model of the dynamic system is a second-order linear-time-invariant system as expressed by Equation (3).

(3) $$\left.\begin{array}{cc} \; & x\left(k\right)=\underset{:=A}{\underset{︸}{\left[\begin{array}{cc} 0 & 1\\ 0.4573 & 0.4654 \end{array}\right]}}x\left(k-1\right)+\underset{:=B}{\underset{︸}{\left[\begin{array}{c} 0.04072\\ 0.03377 \end{array}\right]}}l(k-1)\\ \; & y\left(k\right)=\underset{:=C}{\underset{︸}{\left[\begin{array}{cc} 1 & 0 \end{array}\right]}}x\left(k\right) \end{array}\right\}$$

where *k* is the time index, $x\in R^{2}$ is the state variable, and y∈R and l∈R are the output tension *f* and the input cable length, respectively.

## 3. Control

The number of variables in the knee moment under different terrains makes the task of providing assistance force challenging in soft exoskeleton. Moreover, the deformation of the wraps increases the difficulty in providing robust assistance. This section presents our approach for designing a controller, which is capable of self-adjusting and compensating the error generated due to the variations in the system.

### 3.1. Assistance Strategy

**Figure 5:**
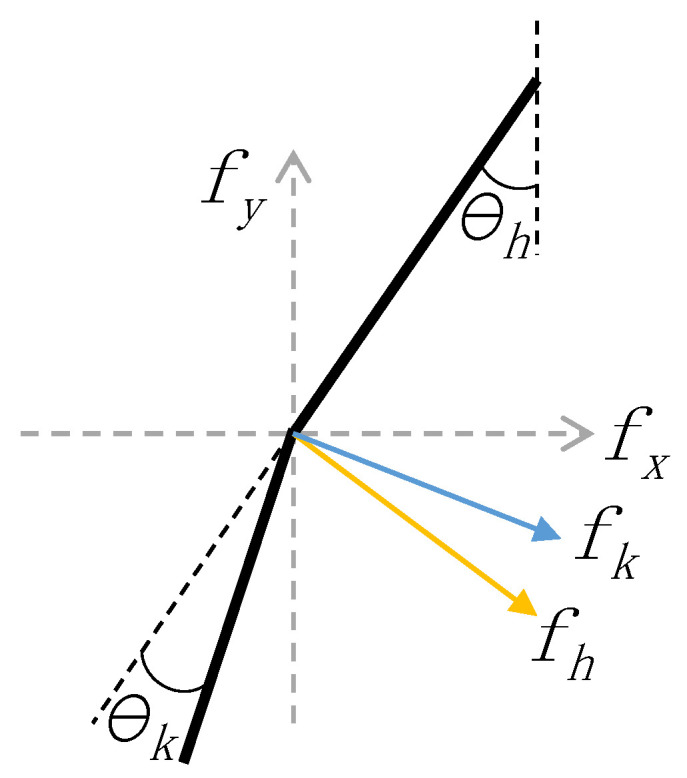
Abstraction of lower limbs and the force in knee board. Where the black line is the abstract of thigh and shank; $f_{x}$ and $f_{y}$ represent the sagittal coordinate system; $f_{h}$ and $f_{k}$ are the forces in the hip and knee joints, respectively; and $\theta_{h}$ and $\theta_{k}$ are the angles of hip and knee joints, respectively.

The moments of flexion and extension in the lower limb joint were analyzed in [29,30]. We constructed three assistance strategies, one for each of uphill, downhill, and level ground scenarios, based on the moments in the hip and knee joints on different terrains. In order to make the assistance strategies applicable in our soft exoskeleton, we maintained the moments in the hip and knee joints as per Section 2. The force on the knee board is shown in Figure 5. Where the black line is the abstract of thigh and shank, $f_{x}$ and $f_{y}$ represent the sagittal coordinate system; $f_{h}$ and $f_{k}$ is the force in hip and knee joints, respectively; and $\theta_{h}$ and $\theta_{k}$ are the angles of hip and knee joint, respectively. The force in the Knee Board can be expressed by Equation (4).

(4) $$f=(-f_{k}\cos\left(\theta_{k}-\theta_{h}\right)+f_{h}\cos\theta_{h},f_{k}\sin\left(\theta_{k}-\theta_{h}\right)-f_{h}\sin\theta_{h})$$

Equation (4) is a vector in the sagittal plane representing the approximated force applied to the Knee Board, which ignores the angle between the Bowden cable and the thigh. The first and second element represent the horizontal component and vertical component of force, respectively. One of the advantages of taking force as vector form is the direction can be easily ignored when constructing assistance strategy later. In Equation (4), $f_{h}$ and $f_{k}$ are the forces in the hip and knee joints which are given by Equations (5) and (6), respectively.

(5) $$\begin{array}{c} f_{h}=\frac{M_{h}}{l_{h}} \end{array}$$

(6) $$\begin{array}{c} f_{k}=\frac{M_{k}}{l_{k}} \end{array}$$

where $M_{h}$ and $M_{k}$ are, respectively, the moments in the hip and knee joints; the values of $M_{h}$, $M_{k}$ $\theta_{h}$, and $\theta_{k}$ in GC are obtained from the work in [30]; and $l_{h}$ and $l_{k}$ are, respectively, the lengths of the thigh and the Knee Board.

**Figure 6:**
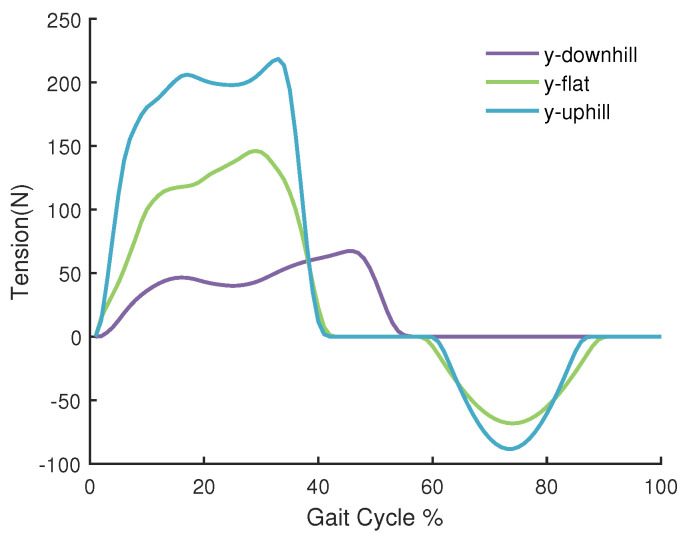
The tension in the Bowden cable at different times in the gait cycle on different slope ground. The positive force represents the force applied in the Knee Board to assist hip joint extension and knee joint extension and the negative force is the force at the front of the thigh, which produces an assistance force in hip joint flexion.

In this paper, we neglect the direction of f, and use its norm as our assistance force in the Knee Board. The assistance force to the frontal thigh was collected by biomechanical moment of hip joint extension [29]. In order to utilize the torque in our system, we refined the raw data by Gaussian Filtering. Finally, the resulting assistance strategy is shown in Figure 6.

### 3.2. Gait Event Estimation Using IMU

The IMUs were attached to the wraps of the thigh, which detected the angular velocity and angular acceleration of the thigh in the sagittal plane. The time of maximum angle of the hip flexion is taken as the starting point of the gait cycle, and the stride time is measured as the time between two consecutive events of maximum hip flexion. Francisco et al. [38] and Zhang et al. [39] have achieved the recognition of human motion and the environmental features through the method of convolutional neural networks (CNN), which proves CNN is a potential method to achieve the classification of terrain. However, this method requires complicated calculations, which is challenging for the processor in this paper. Junwon et al. [40] showed that the gait task can be recognized through the angles of two hip joints at the moment of foot contact. Based on this feature, the terrain identifications can clearly distinguish the terrains of uphill, downhill, and flat ground through the method of support vector machine (SVM).

### 3.3. Controller Design

In this section, we present a POILC-based controller. The length of the Bowden cable used in the soft exoskeleton changes with the movement of the wearer. The slack in the Bowden cable becomes equivalent to adding dead zone nonlinearity to the system, and it reduces the response speed of the soft exoskeleton. We introduced a novel angle feedforward link to compensate the changes in the length of the Bowden cable during walking of the wearer. The POILC approach can eliminate the repetition of disturbance, which directly reduces the error generated due to the difference between the height and position of the wearer. However, POILC is adapted to repetitive and fixed disturbances. In this paper, we take both the POILC and angle feedforward unit as the feedforward link in the feedback controller, which can provide a robust assistance force during walking on different terrains.

**Figure 7:**
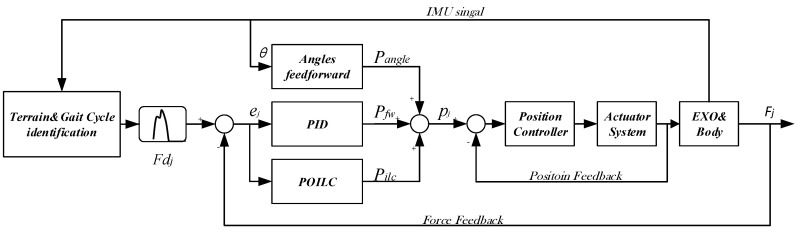
Control architecture with parameter optimal iterative learning control (POILC) and angles feedforward scheme in parallel with PIDfeedback controller.

The control architecture is depicted in Figure 7, where the index *j* denotes iteration of the learning scheme, $Fd_{j}$ denotes the desired force in the *j*th iteration, and $p_{j}$ is the motor position of the controller output, which are constructed by the position of feedback control $p_{\mathrm{fw}}$, POILC feedforward $p_{\mathrm{ilc}}$, and angle feedforward $p_{\mathrm{angle}}$. The relationship between the motor position and the Bowden Cable length is proportional, that is, p=kl. Where the p is the motor position, l is the length of Bowden Cable, and k is the coefficient. The position controller is embedded in motor driver, which can drive the motor to the specified position. $F_{j}$ is the output force from the soft exoskeleton, which feedback to the input terminal through load cell and compare to the input desire force $Fd_{j}$ to get the error $e_{j}$. Then, the hip angle θ in the hip joint is obtained through IMU. The terrain’s type and the gait cycle time can be obtained through the link of terrain and gait cycle identification.

The response time of the control system is dramatically affected by the redundant length of the Bowden cable. The angle feedforward law is applied to eliminate the variation in the length of the Bowden cable during the walking of the wearer, which can be mathematically expressed by Equation (7).

(7) $$p_{\mathrm{angle}}=ak\theta +bI$$

where $I\in R^{2}$ is an identity matrix, $p_{\mathrm{angle}}$ is the position of the motor in two legs, *k* is the coefficient between the length of the Bowden cable and the position of the motor, and *a* and *b* are the same as in Equation (1).

We take a proportional-derivative (PD) controller as our feedback controller, which can be expressed by Equation (8).

(8) $$p_{\mathrm{fw}}=K_{p}e_{j}+K_{d}\Delta e_{j}$$

where $K_{p}\in R^{2\times 2}$ and $K_{d}\in R^{2\times 2}$ denote, respectively, the coefficient matrices of the proportional and derivative controllers, and e and Δe represent, respectively, the errors of the current iteration and its differential. As the system matrix given by Equation (3) is a discrete expression and the norm of A eigenvalues are less than one, the feedback controller has global stability.

The POILC approach is often used to settle the problem with the same batch length, where the batch length means the number of samples in one iteration. In this paper, we set one gait cycle as one iteration, so that the patch length will be changed with the change in the gait cycle under a fixed sampling time. However, it is unavoidable that human gait cycles have variation during walking. We need to regulate the sampling time to ensure that the batch length remains same in each iteration. The sampling time is adjusted at the starting point of the gait cycle, which can be expressed by Equation (9).

(9) $$\begin{array}{c} t_{\mathrm{sample}}=\frac{T}{N} \end{array}$$

where *T* is the gait cycle and *N* is the length of each batch. In our system, N=100.

Similar to that in [41], we apply the parameter optimized approach in our control system. The soft exoskeleton system has two outputs for two legs. For simplicity, we introduce only one side of the POILC controller. The control law is expressed by Equation (10).

(10) $$\begin{array}{c} p_{j+1}\left(t\right)=p_{j}\left(t\right)+\beta_{j+1}e_{j}\left(t+1\right) \end{array}$$

Equation (10) represents a *P*-type ILC control, where the learning rate $\beta_{j+1}$ is to be varied from each iteration. The index *j* denotes the iteration number, and *p* and *e* represent the compensation position and the error, respectively. The learning rate can be computed at the end of the gait cycle through the solution of the quadratic optimization problem as given by Equation (11).

(11) $$\left.\begin{array}{cc} \beta_{j+1} & =\arg\min\{J_{j+1}\left(\beta_{j+1}\right)\}\\ J_{j+1}\left(\beta_{j+1}\right) & =\arg\min\{{∥e_{j+1}∥}^{2}+\omega \beta_{j+1}^{2}\},\omega \geq 0 \end{array}\right\}$$

where ω is the weight parameter, and $\beta_{j+1}$ and $e_{j+1}$ denote the learning rate and the error vector at the (j+1)th iteration, respectively. Where $e_{j+1}$ can be computed through e=r−Gp as given by Equation (12).

(12) $$e_{j+1}=\left(I-\beta_{j+1}G\right)e_{j}$$

where $I\in R^{N\times N}$ is an identity matrix and $G\in R^{N\times N}$ is the Markov parameters of the plant as given by Equation (13).

(13) $$\begin{array}{c} G=\left[\begin{array}{cccc} CB & 0 & \cdots & 0\\ CAB & CB & \cdots & 0\\ \vdots & \vdots & & \vdots \\ CA^{N-1}B & CA^{N-2}B & \cdots & CB \end{array}\right] \end{array}$$

According to the necessary and sufficient conditions of a quadratic optimization problem $dJ/d\beta_{j+1}=0$, we can compute the optimal $\beta_{j+1}$ as expressed by Equation (14).

(14) $$\begin{array}{c} \beta_{j+1}=\frac{\langle e_{j},Ge_{j}\rangle }{\omega +{∥Ge_{j}∥}^{2}} \end{array}$$

The learning rate $\beta_{j+1}$ for the next iteration is computed at the end of the gait cycle. In this way, the convergence speed of the system can be ensured by such a dynamic adjustment. The termination criterion of POILC in our system is that the total error in one gait cycle $e_{j}$ should be less than δ, which is expressed by Equation (15).

(15) $$\begin{array}{c} e_{j}=\sum_{i=1}^{N}e_{j}\left(i\right)<\delta \end{array}$$

where *i* is the index of an element of the error vector and *j* is the index of iteration. In our system, δ=400. If the terrain is not changed during walking, the maximum iterative is set as 300.

## 4. Experimentation

In this section, we discuss the experimental results of our soft exoskeleton. To evaluate the performance of the POILC method in our controller, in the first part we present the effectiveness of tracking the assistance strategy. In the second part, the performance of assistance is presented in the form of the metabolic rate of the wearer.

The control algorithms are executed in a microprocessor and a Raspberry Pi 4B. The Raspberry Pi is used to update the learning rate of POILC and to communicate with the microprocessor through a serial port at a speed of 230,400 bps. The control cycle is selected according to the gait cycle as described in Section 3 and the sample frequency is set as 200 Hz. The experiments were conducted on a treadmill (X9-5918, ShuHua Sports, Shanghai, China) in an indoor environment. The experiment was approved by the Shenzhen Institute of Advanced Integration Technology, and the content of the experiment and its possible impact were explained to all the participants before conducting the experiment.

### 4.1. Model Validation

**Figure 8:**
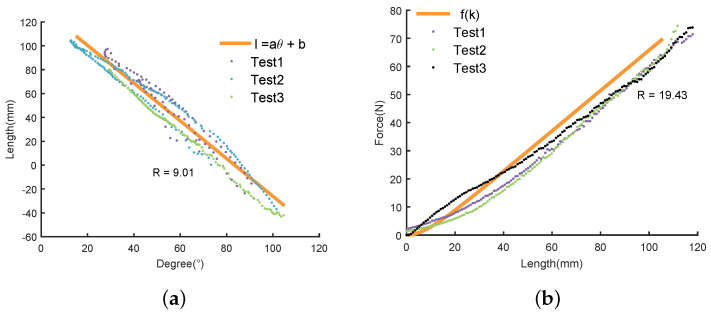
Experimental results of model validation, where R is the root mean square error (RMSE). (**a**) is the result of model (1), where *l* is the length of Bowden Cable. (**b**) is the result of model (2), where *f* is the output force.

The relationship between hip angle and Bowden Cable length is established in Section 2.2, and the system dynamics model is in Section 2.3. In order to validate the model, two experiments were conducted. Model verification in this paper is performed by comparing the predicted and the actual output value. The test data used in model validation is obtained from the experiments in Section 2.2 and Section 2.3. As shown in Figure 8a, model (1) has a satisfying performance. Model (2) is second-order linear time-invariant discrete system, simulated in Simulink of MATLAB under a given input, with sample time set as 0.01 s. As shown in Figure 8b, the output trend of model (2) is similar to that of the actual system.

### 4.2. The Force Tracking Performance Evaluation

To evaluate the performance of force tracking in our soft exoskeleton, we conducted experiments with three healthy adult males (24±1 years old; 66±3 kg weight; 176±5 cm height) walking on a treadmill. The experiments were performed on the treadmill at a speed of 4 km/h. Three slopes of $\pm 10^{\circ }$ and $0^{\circ }$ were set in order to simulate the scenarios of uphill, downhill, and flat ground. The assistance strategies for different terrains have been introduced in Section 3. The subject walked on the treadmill for five minutes at each slope. The tension in the Bowden cable is collected periodically.

**Figure 9:**
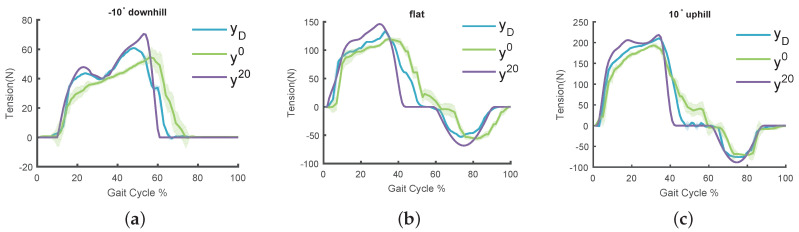
Experimental results of assistance strategy tracking for soft exoskeleton, where (**a**), (**b**) and (**c**) represent the results of downhill, flat, and uphill respectively, $y_{D}$ is the desired assistance force, $y^{0}$ is shows the tracking performance without POILC (no learning, iteration 0), and $y^{20}$ shows the performance improved after applying POILC for 20 iterations.

Figure 9 shows the results of the tracking experiment on different terrains. The tracking performance of the assistance strategy is significantly improved by POILC. Table 2 shows the root mean square error (RMSE) of the tracking results with or without POILC, it demonstrates that the performance of tracking is improved by POILC. Although the wearing position of the soft exoskeleton was not uniform, the results of tracking are found similar to the desired assistance force regardless of the wearing position. The hysteresis of the system response was significantly decreased and the maximum assistance force was increased after 20 iterations. In the control system, we focused mainly on the response time and the stability of the controller, and thus the precision of tracking was relatively low. In our experiment, we also found that very precise tracking is not necessary for the soft exoskeleton control, as the purpose here is to provide real-time and proper assistance to the wearer during walking. The tracking curves showed discontinuity at some points, which might be caused by the nonidentical sampling frequency between the soft exoskeleton system and the host computer. The host computer was used to obtain the actual tension from the load cell of the exoskeleton at the frequency of 200 Hz. The real sampling frequency in the soft exoskeleton system is determined by stride time, and therefore the data is repeated if the real frequency is slow.

### 4.3. Metabolic Cost Test

#### 4.3.1. Experimental Setup and Protocol

Six healthy adult males (24±5 years old, 68±8 kg weight, and 177±6 cm height) participated in the experiment for metabolic rate. We conducted the experiment on a treadmill at a speed of 4 km/h and temperature of $26^{{}^{\circ}}$C. In order to measure the metabolic rate, we used a gas analysis equipment (COSMED K5, Rome, Italy) to record the concentration and volume of the exhaled pulmonary gas, which is mainly composed of carbon dioxide and oxygen. The metabolic rate can also be calculated using the modified Brockway equation [42] as expressed in Equation (16).

(16) $$\begin{array}{c} \Delta H=c_{1}VO_{2}+c_{2}VCO2 \end{array}$$

where coefficients $c_{1}$ and $c_{2}$ are 16.89 and 4.84, respectively, and ΔH is the energy rate (kJ/s). The data of carbon dioxide and oxygen rates were collected at a steady stage during walking.

In order to decrease disturbances to the metabolic rate, the experiments were conducted in two parts, one was training day and another was testing day. The purpose of the training day was to get the subjects familiar with the function of the soft exoskeleton. The main task in the training day was for the subjects to wear the soft exoskeleton and then to walk on the treadmill at three slopes for at least ten minutes at each slope. On the test day, the experiments were walking on a flat ground with or without an exoskeleton, and then conducted two sets of experiments with or without ILC method under wearing exoskeleton. On the another test day, each subject performed six sets of experiments, which were walking in all the three terrains in both cases of wearing and not wearing the soft exoskeleton. As the muscle fatigue has a non-negligible effect on the metabolic consumption, half of an hour was kept between each experiment as a break for the subjects to restore from muscle fatigue. In the experiment test day, the order of each subject’s experiment is random.

**Figure 10:**
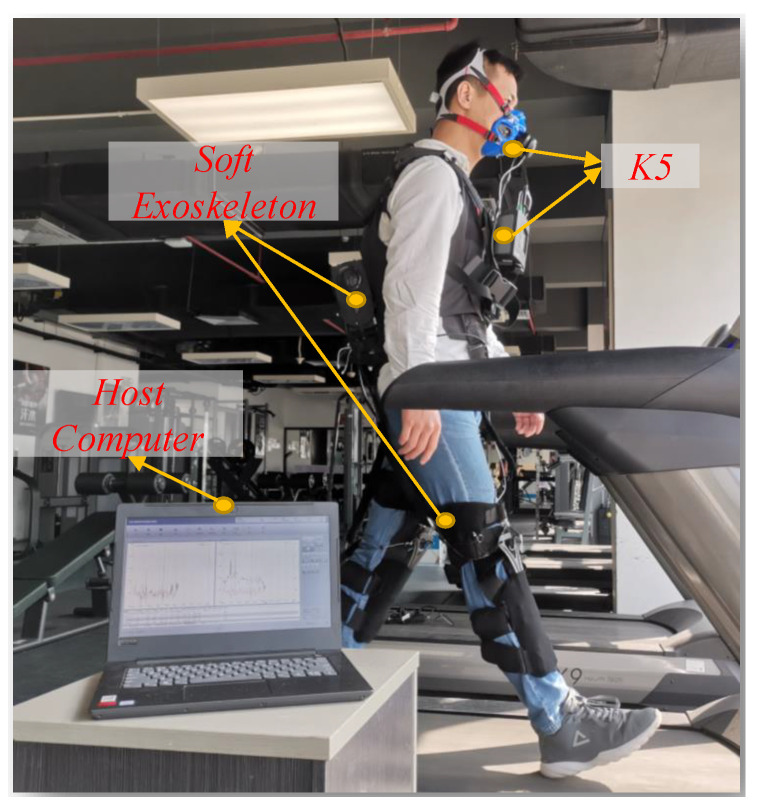
While a subject is walking with the soft exoskeleton, an actuator-driven Bowden cable is used to generate assistance in the lower limb. The metabolic rate is measured through K5.

The duration of each experiment was 15 min, keeping the subjects in standing posture for the first five minutes to measure the standing metabolic rate, and then asked to walk for the remaining ten minutes to obtain the motional metabolic rate. The net metabolic rate was calculated by subtracting the standing metabolic rate from the motional metabolic. Due to the unstable metabolic rate at the beginning of standing and standing-to-walking transitions, the data selected to calculate the metabolism of standing and walking are the last 3 and 5 min of data of the standing and walking phases, respectively. The experimental progress is shown in Figure 10.

#### 4.3.2. Metabolic Reduction by POILC

**Figure 11:**
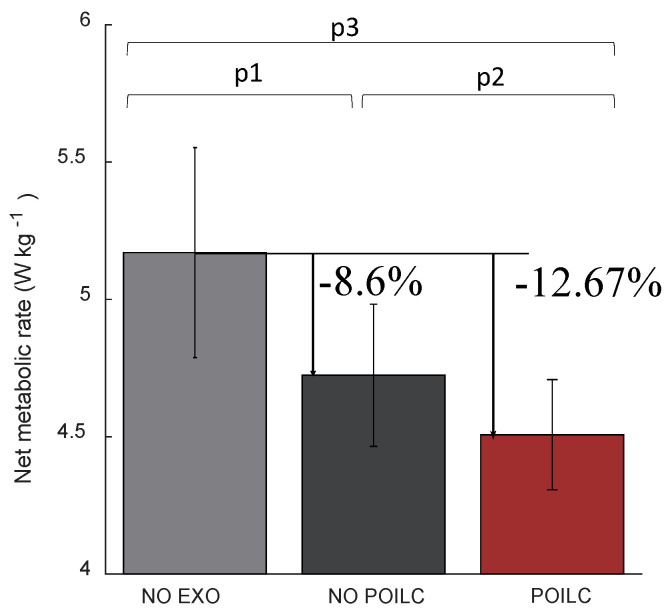
The metabolic reduction when walking on the flat ground. The NO EXO, NO ILC, and ILC present the case of not wearing soft exoskeleton, and wearing exoskeleton without and with POILC method. p1, p2, and p3 are the result of two-side t-tests, which are 0.002, 0.134, and 0.006, respectively.

The tracking error of the desired force is decreased by adding the POILC method. In order to verify the performance improvement of the soft exoskeleton due to the reduction in tracking error, a comparative test is conducted on flat ground. The reduction in metabolic rates in the condition of with ILC and without is as shown in Figure 11. The results demonstrate that the performance of the soft exoskeleton is improved by reducing the tracking error. The net metabolism is reduced more when the POILC method is applied. The net metabolism decreased by 8.6% without POILC method and 12.6% with POILC method, respectively.

#### 4.3.3. Metabolic Reduction in Three Slopes

The metabolic rates of the subjects in each experiment are shown in Table 3. The results demonstrate that the metabolic rate was highest in the uphill mode and lowest in the downhill mode, as predicted. The reductions in the metabolic rate in the downhill and flat ground modes were considerably similar, which was dramatically different from the case of uphill. The average reductions in the net metabolic rate were 9.82%, 12.84%, and 21.71% compared to the case of not wearing the soft exoskeleton in the downhill, flat ground, and uphill, respectively, where the absolute reductions were 0.28 W/kg, 0.72 W/kg, and 1.60 W/kg, respectively.

**Figure 12:**
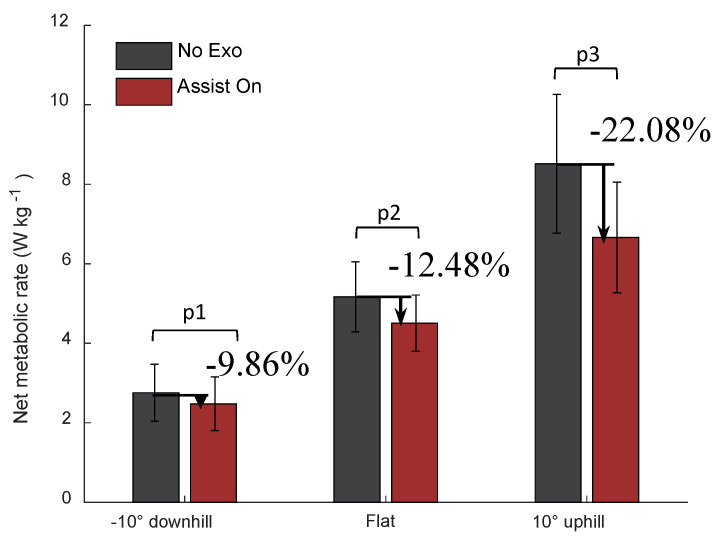
Metabolic rate from the average of three participant experiments for combined different terrains comparison between wearing the soft exoskeleton for assistance (Assist On) and not wearing it (No Exo). p1, p2, and p3 are the results of two-sided t-tests, which are 0.047, 0.051, and 0.038, respectively.

In order to clearly visualize the amount of reduction in metabolic rate upon wearing the soft exoskeleton, we plot a bar chart comparing the differences as shown in Figure 12.

The results demonstrate that the metabolic rate decreases in three terrains, and the decrease in the uphill is dramatically, which is consistent to the fact that the knee moment increases in the uphill walking. It also verifies our assumption that an assistance to the knee joint is advantageous for uphill walking.

## 5. Discussion

This work presents the design of a novel soft exoskeleton for lower limb joints, which can provide assistance for hip joint extension and flexion and for knee joint extension. We built three assistance profiles for three differently sloped terrains, and designed a controller based on the POILC method and feedforward model. The performance of the control strategy and the net metabolic costs in different slopes are evaluated.

The net metabolic reduction is related to the assistance to the joint in the lower limb. An assistance to the hip extension can reduce the metabolic rate by 9.3% in [43], and that to the ankle joint can reduce the same by 24.72% in comparison to those without any assistance [33]. An intuitive idea is that the metabolic rate will be decreased dramatically if we assist multiple joints. The authors of [8] claimed that assistance torques in the ankle and hip joints are beneficial to reduce the net metabolic rate, which was demonstrated by reducing the metabolic rate of walking by 16.93% in comparison to the condition of wearing an exosuit without any external power. McIntosh et al. [30] demonstrated that the moment of the knee joint varies with slopes. Taking a reduced torque to the knee joint and combining with the advantage of the multi-joint assistance as our motivation, we designed a multi-joint assistance soft exoskeleton to reduce the metabolic of walking on different slopes. Taking the advantage of the multiple joint assistance, we designed a soft exoskeleton for providing assistance to both hip and knee joints so as to reduce the metabolic consumption of walking on different slopes. The final result demonstrates the feasibility of our scheme of assisting the hip and knee. The metabolic rate decreased in three differently sloped terrains, and the greatest reduction is found when walking on uphill terrain. The maximum metabolic rate of walking on a $10^{\circ }$ slope is reduced by 22.08% compared to the condition of not wearing the soft exoskeleton.

As the biological torque in the lower limb joint will change with the terrain, the same assistance strategy is likely to have different effects on metabolic rate. The influences of the assistance time and the peak force on the reduction of the metabolic rate were studied in [44,45]. In this paper, we build three assistance strategies, based on the biological moments in the hip and knee joints, for three terrain slopes such as $-10^{\circ }$ downhill, flat, and $10^{\circ }$ uphill. These assistance schemes have varied effects on different terrains. However, any specific relationship between the assistance strategy and the slope of terrain is not yet developed. We can construct more terrains with an attempt to develop a more effective assistance strategy in future work. Apart from biological torques, the human in loop method is also an alternative to determine the proper assistance strategy, which has been used in [31,33] to optimize the assistance force for improving the performance in reducing the metabolic rate.

In this paper, multiple joints on a leg were actuated by a single motor, which significantly reduced the weight of our soft exoskeleton system and then decreased the metabolic consumption caused by the weight of the system. We have a system weight of only 4869 g, which is lighter than most portable soft exoskeletons. Table 4 shows the result of a comparison with some mainstream soft exoskeletons, where N.A. means that the system is not portable. By comparison, we found that our system has a lighter weight and can help wearers save more energy cost on different slopes. The disadvantage of this scheme is that the motor suffers from the reversion with a high frequency. However, the motor is required to reverse its rotation in a short period of time when the assistance force switches from the Knee Board to the front side of the thigh, while still keeping the Bowden cable tightened to generate a real-time assistance force. The reversing time is shortened with increasing walking speed. The compatible maximum walking speed of the motor at that point of time was not studied. It would be considered in future as a limiting factor of the scenario of running.

The difference between the wearing position of the soft exoskeleton and the height of the wearer increases the challenge of the soft exoskeleton to provide an accurate assistance force to walking. Ding et al. [26] solved this problem by iteratively adjusting the position of the actuator motor, where the adjusted value is determined according to the previous error of tracking. In this paper, this challenge is dealt with by introducing the POILC. The control system is capable of adapting quickly to the biological features of the wearer and to compensate the error caused due to the deformation of the wraps and the wearing position. However, in our experiment we find that the performance of tracking with POILC is comparable to that reported in [21]. A possible reason is that the structure of our assistance strategy is more complex than those of earlier models. Another reason is that the error is likely to occur from the tension detection when the assistance switches from the Knee Board to the front side of the thigh. There is a tiny jitter of tension in the Bowden cable when the heel strikes, which may also cause the tracking error. The influence of this error upon increasing the walking speed or the assistance force shall be studied in future.

## 6. Conclusions

The important purpose of the soft exoskeleton is to decrease the energy consumption of users by providing assistance and restricting the metabolic penalty due to the weight of the system. Walking on different terrains leads to different biomechanics. However, researchers rarely study assistance force on uneven terrains. In this paper, we designed a novel assistance scheme to improve the performance of an exoskeleton system, which simultaneously provides assistance to hip joint flexion and extension and knee joint extension. We built three assistance strategies for three slopes based on the biological moment of human walking on corresponding terrains, and introduced POILC to improve the tracking performance of the system. The POILC in our controller reduces the error generated due to the difference between the wearing position and biological features of the wearer, which is also verified by the results of the tracking experiment. We conducted a series of experiments to validate that our novel scheme is beneficial in providing assistance torque on different terrains. The results show that the reductions obtained in the net metabolic rate in the downhill, flat ground, and uphill terrains are, respectively, 9.86%, 12.48%, and 22.08% compared to the condition of not wearing the soft exoskeleton. The corresponding absolute reductions in the metabolic rate were 0.27 W/kg, 0.72 W/kg, and 1.60 W/kg, respectively.

The results of this study show that multi-joint assistance is beneficial to reduce net metabolic rate during walking. Further, combined with the knee joint assistance, the metabolic rate in the uphill terrain can be reduced dramatically. Due to the light weight of the exoskeleton, the assistance achieved satisfactory performance and adaptation to different terrains; the soft exoskeleton in this paper is suitable to provide assistance for health people, especially for the older adults. The performance of the assistance on a complex terrain and the corresponding assistance strategy shall be studied in future work.

## Figures and Tables

**Table 1:** The weight of each part of our soft exoskeleton.

Device	Number	Mass (g)
motor	2	662
actuator	2	320
gearbox	2	768
pulley	2	220
battery	1	700
wraps	2	630
vest	1	670
IMU	2	80
load cell	4	188
other part	–	621
total	–	4859

**Table 2:** The RMSE in different slopes with POILC and without POILC, where $y^{0}$ and $y^{20}$ are the tracking results with and without POILC, respectively.

Terrain	$y^{0}$	$y^{20}$	Reduction
$-10^{\circ }downhill$	13.55	6.61	51.22%
flat	37.20	19.92	46.45%
$10^{\circ }uphill$	37.61	25.20	33.00%

**Table 3:** Metabolic testing results of the downhill, flat, and uphill modes. Where NE and AO represent the no exoskeleton and the assist on modes, respectively, which means subjects were either not wearing the exoskeleton or wearing exoskeleton, Re represents the reduction.

Subject	Downhill (−10°)			Flat (0°)			Uphill (10°)		
	NE	AO	Re	NE	AO	Re	NE	AO	Re
	(W/kg)	(W/kg)		(W/kg)	(W/kg)		(W/kg)	(W/kg)	
S1	2.88	2.67	7.42%	4.69	4.20	10.61%	7.48	5.79	22.54%
S2	2.60	2.36	9.11%	5.42	4.72	12.83%	7.54	5.93	21.41%
S3	2.67	2.37	11.57%	5.90	5.08	13.90%	9.53	7.27	21.51%
S4	2.73	2.48	9.30%	6.44	5.58	13.34%	6.50	5.05	22.35%
S5	2.98	2.62	11.84%	5.89	5.29	10.20%	6.42	5.37	16.47%
S6	3.20	2.88	9.91%	6.19	5.32	14.00%	5.85	4.33	25.99%

**Table 4:** Comparison with popular soft exoskeletons. Where N.A. means that the the system is not portable.

Research	Assistance	System Weight (Kg)	Application Scenarios	Maximum Energy Cost Reduction (%)
Kim [43]	Hip extension	5.004	Different slope	9.3
Sangjun [8]	Hip extension and flexion	5.1	Outdoors	16.93
	& Ankle plantar flexion			
Ding [31]	Hip extension	N.A	Flat ground	17.4
Juanjuan Zhang [33]	Ankle plantar flexion	N.A	Flat ground	24.2
Collins [46]	Ankle plantar flexion	1	Flat ground	7.2
This work	Hip extension and flexion	4.6	Different slope	22.08
	& Knee extension			

## Abbreviations

The following abbreviations are used in this manuscript.

GC	Gait Cycle
IMU	Inertial measurement unit
ILC	Iterative learning control
POILC	Parameter optimal iterative learning control

## References

[1] Winter D.A., Patla A.E., Frank J.S., Walt S.E. (1990). Biomechanical Walking Pattern Changes in the Fit and Healthy Elderly. Phys. Ther..

[2] Duschau-Wicke A., Zitzewitz J.V., Caprez A., Lunenburger L., Riener R. (2010). Path control: A method for patient-cooperative robot-aided gait rehabilitation. IEEE Trans. Neural Syst. Rehabil. Eng..

[3] Wehner M., Quinlivan B., Aubin P.M., Martinez-Villalpando E., Baumann M., Stirling L., Holt K., Wood R., Walsh C. A lightweight soft exosuit for gait assistance. Proceedings of the 2013 IEEE International Conference on Robotics and Automation.

[4] Caldwell D.G., Tsagarakis N.G., Kousidou S., Costa N., Sarakoglou I. (2007). “SOFT” Exoskeletons for upper and lower body rehabilitation—Design, control and testing. Int. J. Humanoid Robot..

[5] Schmidt K., Duarte J.E., Grimmer M., Sancho-Puchades A., Wei H., Easthope C.S., Riener R. (2017). The myosuit: Bi-articular anti-gravity exosuit that reduces hip extensor activity in sitting transfers. Front. Neurorobotics.

[6] Asbeck A.T., Schmidt K., Walsh C.J. (2015). Soft exosuit for hip assistance. Robot. Auton. Syst..

[7] Asbeck A.T., Rossi S.M.M.D., Holt K.G., Walsh C.J. (2015). A biologically inspired soft exosuit for walking assistance. Int. J. Robot. Res..

[8] Lee S., Karavas N., Quinlivan B.T., LouiseRyan D., Perry D., Eckert-Erdheim A., Murphy P., Goldy T.G., Menard N., Athanassiu M. Autonomous multi-joint soft exosuit for assistance with walking Overground. Proceedings of the 2018 IEEE International Conference on Robotics and Automation (ICRA).

[9] Ortiz J., Rocon E., Power V., de Eyto A., O’Sullivan L., Wirz M., Bauer C., Schülein S., Stadler K.S., Mazzolai B. (2017). Xosoft-a vision for a soft modular lower limb exoskeleton. Wearable Robotics: Challenges and Trends.

[10] Ortiz J., Di Natali C., Caldwell D.G. (2018). XoSoft-iterative design of a modular soft lower limb exoskeleton. Proceedings of the International Symposium on Wearable Robotics.

[11] Park Y.L., Santos J., Galloway K.G., Goldfield E.C., Wood R.J. A soft wearable robotic device for active knee motions using flat pneumatic artificial muscles. Proceedings of the 2014 IEEE International Conference on Robotics and Automation (ICRA).

[12] Collins S.H., Kim M., Chen T., Chen T. An ankle-foot prosthesis emulator with control of plantarflexion and inversion-eversion torque. Proceedings of the 2015 IEEE International Conference on Robotics and Automation (ICRA).

[13] Sasaki D., Noritsugu T., Takaiwa M. Development of pneumatic lower limb power assist wear driven with wearable air supply system. Proceedings of the 2013 IEEE/RSJ International Conference on Intelligent Robots and Systems.

[14] Di Natali C., Sadeghi A., Mondini A., Bottenberg E., Hartigan B., De Eyto A., O’Sullivan L., Rocon E., Stadler K., Mazzolai B. (2020). Pneumatic Quasi-Passive Actuation for Soft Assistive Lower Limbs Exoskeleton. Front. Neurorobotics.

[15] Jin S., Iwamoto N., Hashimoto K., Yamamoto M. (2016). Experimental evaluation of energy efficiency for a soft wearable robotic suit. IEEE Trans. Neural Syst. Rehabil. Eng..

[16] Di Natali C., Poliero T., Sposito M., Graf E., Bauer C., Pauli C., Bottenberg E., De Eyto A., O’Sullivan L., Hidalgo A.F. (2019). Design and evaluation of a soft assistive lower limb exoskeleton. Robotica.

[17] Manti M., Cacucciolo V., Cianchetti M. (2016). Stiffening in soft robotics: A review of the state of the art. IEEE Robot. Autom. Mag..

[18] Lenzi T., Carrozza M.C., Agrawal S.K. (2013). Powered hip exoskeletons can reduce the user’s hip and ankle muscle activations during walking. IEEE Trans. Neural Syst. Rehabil. Eng..

[19] Asbeck A.T., Schmidt K., Galiana I., Wagner D., Walsh C.J. Multi-joint soft exosuit for gait assistance. Proceedings of the 2015 IEEE International Conference on Robotics and Automation (ICRA).

[20] Ding Y., Galiana I., Asbeck A., Quinlivan B., De Rossi S.M.M., Walsh C. Multi-joint actuation platform for lower extremity soft exosuits. Proceedings of the 2014 IEEE International Conference on Robotics and Automation (ICRA).

[21] Lee G., Ding Y., Bujanda I.G., Karavas N., Zhou Y.M., Walsh C.J. Improved assistive profile tracking of soft exosuits for walking and jogging with off-board actuation. Proceedings of the 2017 IEEE/RSJ International Conference on Intelligent Robots and Systems (IROS).

[22] Bristow D.A., Tharayil M., Alleyne A.G. (2006). A survey of iterative learning control. IEEE Control Syst. Mag..

[23] Angelini F., Della Santina C., Garabini M., Bianchi M., Gasparri G.M., Grioli G., Catalano M.G., Bicchi A. (2018). Decentralized trajectory tracking control for soft robots interacting with the environment. IEEE Trans. Robot..

[24] Marchese A.D., Tedrake R., Rus D. (2016). Dynamics and trajectory optimization for a soft spatial fluidic elastomer manipulator. Int. J. Robot. Res..

[25] Hofer M., Spannagl L., D’Andrea R. (2019). Iterative Learning Control for Fast and Accurate Position Tracking with a Soft Robotic Arm. arXiv.

[26] Ding Y., Galiana I., Siviy C., Panizzolo F.A., Walsh C. IMU-based iterative control for hip extension assistance with a soft exosuit. Proceedings of the 2016 IEEE International Conference on Robotics and Automation (ICRA).

[27] Huang R., Cheng H., Guo H., Chen Q., Lin X. Hierarchical interactive learning for a human-powered augmentation lower exoskeleton. Proceedings of the 2016 IEEE international conference on robotics and automation (ICRA).

[28] Li J.A., Dong D., Wei Z., Liu Y., Pan Y., Nori F., Zhang X. (2020). Quantum reinforcement learning during human decision-making. Nat. Hum. Behav..

[29] Perry J., Davids J.R., Jon R. (1992). Gait analysis: Normal and pathological function. J. Pediatr. Orthop..

[30] McIntosh A.S., Beatty K.T., Dwan L.N., Vickers D.R. (2006). Gait dynamics on an inclined walkway. J. Biomech..

[31] Ding Y., Kim M., Kuindersma S., Walsh C.J. (2018). Human-in-the-loop optimization of hip assistance with a soft exosuit during walking. Sci. Robot..

[32] Voloshina A.S., Kuo A.D., Daley M.A., Ferris D.P. (2013). Biomechanics and energetics of walking on uneven terrain. J. Exp. Biol..

[33] Zhang J., Fiers P., Witte K.A., Jackson R.W., Poggensee K.L., Atkeson C.G., Collins S.H. (2017). Human-in-the-loop optimization of exoskeleton assistance during walking. Science.

[34] Felt W., Selinger J.C., Donelan J.M., Remy C.D. (2015). “Body-In-The-Loop”: Optimizing Device Parameters Using Measures of Instantaneous Energetic Cost. PLoS ONE.

[35] Haufe F.L., Wolf P., Riener R. (2020). Human-in-the-loop optimization of a multi-joint wearable robot for movement assistance. Proc. Autom. Med Eng..

[36] Wu X., Fang K., Chen C., Zhang Y. (2020). Development of a lower limb multi-joint assistance soft exosuit. Sci. China Inf. Sci..

[37] Knapik J.J., Reynolds K.L., Harman E. (2004). Soldier load carriage: Historical, physiological, biomechanical, and medical aspects. Mil. Med..

[38] Ordóñez F.J., Roggen D. (2016). Deep convolutional and lstm recurrent neural networks for multimodal wearable activity recognition. Sensors.

[39] Zhang K., Xiong C., Zhang W., Liu H., Lai D., Rong Y., Fu C. (2019). Environmental features recognition for lower limb prostheses toward predictive walking. IEEE Trans. Neural Syst. Rehabil. Eng..

[40] Jang J., Kim K., Lee J., Lim B., Shim Y. Online gait task recognition algorithm for hip exoskeleton. Proceedings of the 2015 IEEE/RSJ International Conference on Intelligent Robots and Systems (IROS).

[41] Owens D.H., Hätönen J. (2005). Iterative learning control—An optimization paradigm. Annu. Rev. Control.

[42] Brockway J. (1987). Derivation of formulae used to calculate energy expenditure in man. Hum. Nutr. Clin. Nutr..

[43] Kim J., Lee G., Heimgartner R., Revi D.A., Karavas N., Nathanson D., Galiana I., Eckert-Erdheim A., Murphy P., Perry D. (2019). Reducing the metabolic rate of walking and running with a versatile, portable exosuit. Science.

[44] Ding Y., Panizzolo F.A., Siviy C., Malcolm P., Galiana I., Holt K.G., Walsh C.J. (2016). Effect of timing of hip extension assistance during loaded walking with a soft exosuit. J. Neuroeng. Rehabil..

[45] Lee J., Seo K., Lim B., Jang J., Kim K., Choi H. Effects of assistance timing on metabolic cost, assistance power, and gait parameters for a hip-type exoskeleton. Proceedings of the 2017 International Conference on Rehabilitation Robotics (ICORR).

[46] Collins S.H., Wiggin M.B., Sawicki G.S. (2015). Reducing the energy cost of human walking using an unpowered exoskeleton. Nature.

